# A comparison of shot selection and goal scoring between collegiate and professional women’s and men’s ice hockey

**DOI:** 10.3389/fspor.2025.1648099

**Published:** 2025-10-20

**Authors:** Ben Csiernik, Mikaeli Cavell, Kassidy Nauboris, Nick Wattie

**Affiliations:** ^1^ Faculty of Health Sciences, Ontario Tech University, Oshawa, ON, Canada; ^2^ Athletics Department, Ontario Tech University, Oshawa, ON, Canada

**Keywords:** performance, sport, tactics, behavior, strategy

## Abstract

The purpose of this study was to examine the differences and similarities in shooting and scoring trends between collegiate women's hockey, and professional men's and women's hockey. A multinomial logistic regression using shot data from the Ontario University Athletics women's hockey league, Professional Women's Hockey League, and the National Hockey League revealed statistically significant differences, indicating that each league has unique shooting patterns. Understanding that each league has different shooting and scoring profiles is relevant to coaches, who may benefit from using normative data from their own leagues to support decision making, rather than results from other leagues.

## Introduction

With increased data availability, advanced analytics have become an integral part of high level sport (1, 2). Despite analytics being used among professional organizations and conducted in public forums (i.e., blogs), research in ice hockey has lagged behind relative to other more global sports. Currently, the National Hockey League (NHL) and the Professional Women's Hockey League (PWHL) record event tracking data, including shot location and outcomes, that are accessible via an application programming interface, making some event data accessible to the public. Importantly, limitations surrounding data availability has made it difficult to analyze ice hockey at the professional level to the same degree as other sports. For example, publicly available tracking data in football that is not available in ice hockey currently includes passing events, allowing for greater opportunities for analysis (3). These differences are further compounded when analyzing sub-elite level sport (i.e collegiate ice hockey), where all event data must be manually recorded as no publicly available data exists.

To date, when comparing sub-elite athletes to professional ice hockey athletes, most research has focused on physiological differences (4, 5) rather than tactical considerations and approaches. While this is undoubtedly important, it is equally as important for coaches to understand the skill and tactical differences that exist between these groups so they can appropriately guide athlete development, and team performance. Previous work examining men's and women's football has identified that notable differences exist in shooting and scoring behavior (6, 7). Specifically, research has shown that in professional football, women have distinctive shooting and scoring patterns when compared to men, where women have a greater shooting conversion percentage, score more goals per game, and take shots with higher quality by shooting from closer to the net and the midline of the pitch (6, 7). Despite this line of inquiry seen in football, limited research exists examining this difference in ice hockey (8). Therefore, the purpose of this study is to examine the similarities and differences in shooting and scoring patterns between collegiate women's hockey, professional women's hockey, and professional men's hockey.

## Methods

Shot information, including the X and Y location of the shot, and the shot outcome, was collected for three leagues: the Ontario University Athletics (OUA) women's hockey league, the Professional Women's Hockey League (PWHL), and the NHL. No data from men's collegiate hockey was included due to a lack of data availability. Unblocked shot attempts, which include shot attempts stopped by the goaltender, or are shot wide of the net, from the OUA were recorded from games between 2023 and 2025. Unblocked shot attempts from the PWHL were retrieved from Gilles Dignard's public database from the 2023–2024 season^1^, and unblocked shot attempts from the NHL were retrieved from MoneyPuck.com for the 2023–2024 season^2^. Given that the NHL features more teams and games per year, a sample of NHL games matching the number of OUA games was used. All shot attempts towards an empty net (with no goaltender currently on the ice) were excluded from the analysis, as shot behavior is modified by the absence of a defending goaltender. Additionally, all shots originating from below the goal line were excluded due to uncertainty surrounding how shot data was collected. Lastly, all shot data across the three leagues includes all even strength, power play, and penalty kill opportunities.

**Figure 1:**
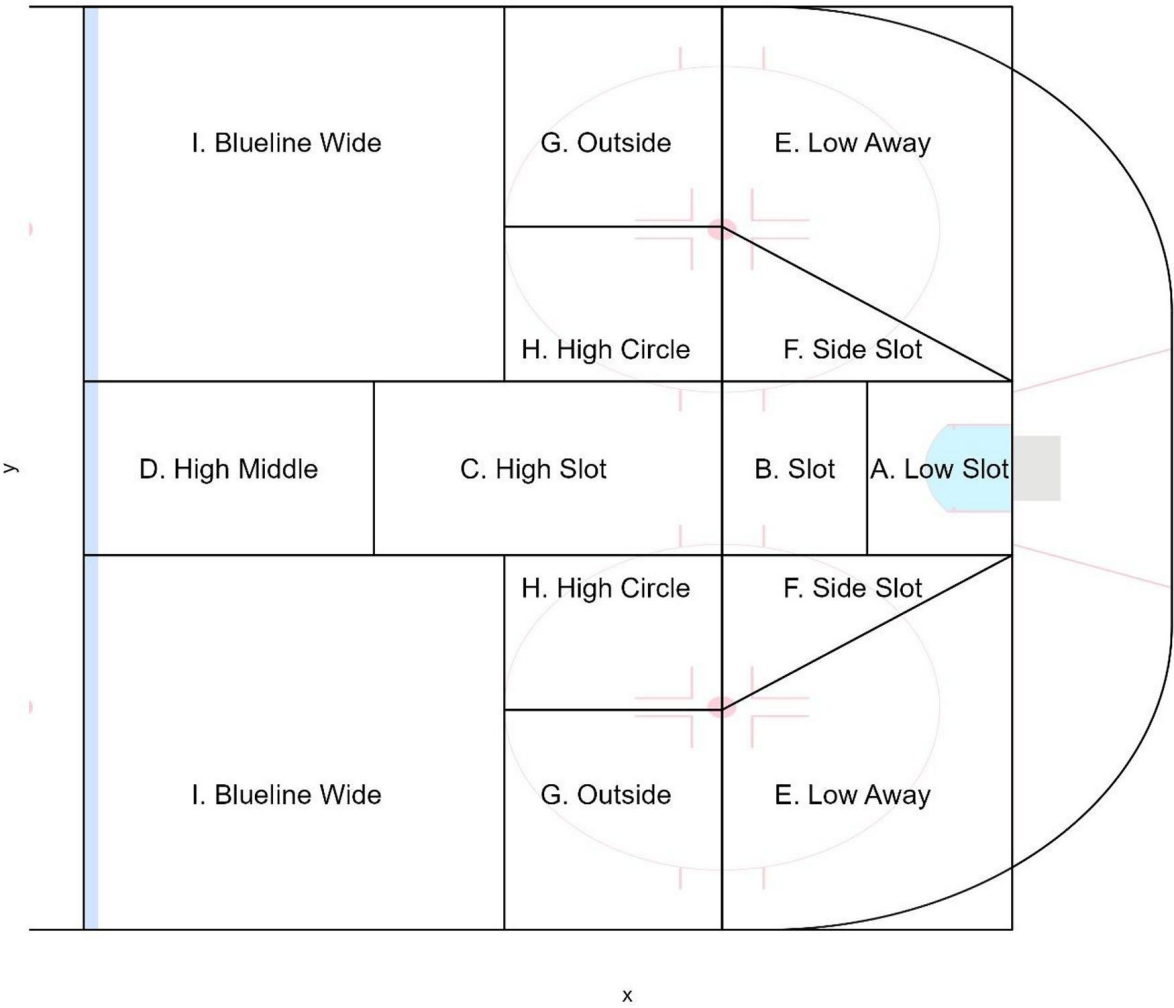
Scoring zone chart.

To compare shooting patterns, shot attempts from each league were binned into one of nine scoring zones (Figure 1), similar to original zones created by WAR on Ice^3^. These zones were originally created based on goal scoring frequencies in the NHL. Using these nine categories (which collapse left and right zones of the ice), the number of shot attempts occurring in each zone was totaled for each team in every game. In total, shots from 192 complete games were analyzed from the OUA and NHL, and 72 games from the PWHL. To compare league shooting similarities, a multinomial logistic regression was used where the league was the dependent variable, and each shooting zone was used as independent variables. Checking model assumptions revealed independence between variables and leagues, and predictive probabilities correctly identified games belonging to the NHL 64.8% of the time, the OUA 69.8% of the time, and the PWHL 53.% of the time. Results were interpreted using marginal effects to allow for the comparison of shot patterns between leagues by zone. In each case, summing the marginal effects of each unique shot zone across the three leagues results in a value of 0. Therefore, leagues showing nearly identical shooting trends would have similar marginal effects. In the case where no differences existed across the three leagues in a scoring zone, the marginal effect would be near zero for each league. After Bonferroni adjustment was applied to account for multiple tests across zones, the statistical significance threshold was *p* < .002. Marginal effects were created using the “marginaleffects” package in R, while all data cleaning and visualizations were conducted using the “tidyverse” package using R statistical software (9–11). This project received research ethics exemption from Ontario Tech University (#18783).

## Results

In total, 6,088 PWHL shot attempts, 15,930 NHL shot attempts, and 14,078 OUA shot attempts (13,978 included in the regression analysis), were included. The number of shots and goals seen per 1,000 unblocked shot attempts, as well as the results from the multinomial logistic regression model are found in Table 1.

**Table 1:** Goals and unblocked shot attempts by zone per 1,000 unblocked shot attempts, and marginal effects.

Scoring zone	NHL	PWHL	OUA
A—Low slot	19.77 goals	10.84 goals	24.30 goals
	126 shots	58 shots	131 Shots
	0.035**	−0.047	0.012**
B—Slot	9.73 goals	11.00 goals	10.44 goals
	75 shots	64 shots	83 shots
	0.010	−0.085	−0.001
C—High slot	8.03 goals	5.91 goals	5.54 goals
	68 shots	108 shots	97 shots
	−0.057**	0.029**	0.028**
D—High middle	1.51 goals	1.81 goals	0.71 goals
	39 shots	42 shots	30 shots
	0.035	0.024	−0.059**
E—Low away	4.39 goals	4.77 goals	2.27 goals
	127 shots	136 shots	108 shots
	0.024**	0.012	−0.037**
F—Side slot	9.42 goals	4.77 goals	7.53 goals
	89 shots	79 shots	109 shots
	−0.008	−0.015*	0.023**
G—Outside	2.95 goals	1.48 goals	0.71 goals
	109 shots	97 shots	90 shots
	0.034**	−0.003	−0.031**
H—High circle	8.98 goals	6.40 goals	4.97 goals
	118 shots	153 shots	123 shots
	−0.007	0.027**	−0.020**
I—Blueline wide	5.65 goals	5.11 goals	2.97 goals
	249 shots	263 shots	229 shots
	0.015**	0.006	−0.021**
Total	70.43 goals	52.09 goals	59.44 goals
	1,000 shots	1,000 shots	1,000 shots

*P*-values: **p < 0.001, *p < 0.002.

The results from the multinomial logistic regression model, and the adjusted goal and shooting rates, highlight that there are distinctive shot patterns exhibited across the three leagues examined. Across the leagues, a number of shooting zones demonstrated statistically significant differences, indicating that shot volume differs by league across the zones. When comparing the OUA with the PWHL in contrast to the NHL, only the high slot zone shared similar marginal effects between the two women's leagues relative to the men's league.

The number of goals and shots from each league and shooting zone per 1,000 unblocked shot attempts can also be found in Table 1. Despite differences in shot volume patterns, the OUA and the PWHL shared similar scoring characteristics in the High Slot, and demonstrated similar shooting percentages in the High Slot, the Side Slot, and the High Circle (Table 2). In these specific zones, the OUA and the PWHL took a similar, or much greater number of unblocked shots from these areas relative to the NHL, despite scoring approximately 50% less from these same areas, highlighting a difference in shooting efficiency in the NHL relative to the two women's leagues. Additionally, both the percentage of shots from each zone (shot share), and the percentage of goals for each zone (goal share), can be found by league in Table 3. In each case, summing the shot share, and goal share from each zone will total to 100 percent rounded, accounting for all shots in the datasets.

**Table 2:** Shooting percentage, and women's leagues shooting percentage ratio to NHL.

Zone	NHL	PWHL	OUA
A—Low slot	15.73%	18.70%	18.54%
		1.18	1.18
B—Slot	13.07%	17.18%	12.56%
		1.31	0.96
C—High slot	11.81%	5.47%	5.71%
		0.46	0.48
D—High middle	3.84%	4.35%	2.37%
		1.13	0.62
E—Low away	3.45%	3.52%	2.11%
		1.02	0.61
F—Side slot	10.62%	6.02%	6.87%
		0.57	0.65
G—Outside	2.71%	1.52%	0.79%
		0.56	0.29
H—High circle	7.60%	4.18%	4.04%
		0.55	0.53
I—Blueline wide	2.27%	1.94%	1.30%
		0.85	0.57

**Table 3:** Shot share, and goal share by league and zone.

Zone	NHL	PWHL	OUA
A—Low slot	12.57%	5.80%	13.11%
	28.07%	20.82%	40.86%
B—Slot	7.44%	6.41%	8.31%
	13.81%	21.14%	17.56%
C—High slot	6.80%	10.81%	9.70%
	11.40%	11.36%	9.32%
D—High middle	3.92%	4.16%	3.00%
	2.14%	3.47%	1.19%
E—Low away	12.74%	13.55%	10.78%
	6.24%	9.15%	3.82%
F—Side slot	8.86%	7.92%	10.96%
	13.37%	9.15%	12.66%
G—Outside	10.89%	9.74%	8.97%
	4.19%	2.84%	1.19%
H—High circle	11.81%	15.31%	12.30%
	12.74%	12.30%	8.36%
I—Blueline wide	24.94%	26.31%	22.87%
	8.02%	9.78%	5.02%

## Discussion

The purpose of this study was to empirically compare shooting and goal scoring between collegiate women's hockey, professional women's hockey, and professional men's hockey. As demonstrated in Table 1, while some similarities existed between leagues, there are clear differences in shot location, and shooting and goal scoring patterns across the three leagues. The results from this analysis demonstrate that both shot opportunity and goal scoring differ between the men's and women's leagues, suggesting that specific considerations are necessary on a per league basis.

Overall, differences exist between leagues regardless of the measurement used to evaluate shooting behavior, including shooting and scoring frequencies, shot share, goal share, and shooting percentage. Beyond the significant differences between the three leagues, an interesting trend emerged in goal scoring and its potential downstream impact on shooting behavior. In the High Slot, Side Slot, and High Circle, men's professional hockey players demonstrated a shooting percentage that was at least 54% greater (range 54%–116%) than that of the two women's leagues. For example, NHL players scored on 11.81% of their shots in the High Slot, while only 5.47% and 5.71% of shots from that same zone were goals in the PWHL and OUA, respectively. Interestingly, with the exception of the Side Slot area for the PWHL, the women's hockey leagues took more shots from those zones than NHL players, despite the notable difference in shooting percentage. While the reason for this trend is unclear, plausible explanations exist. First, it may be possible that the defensive approaches seen in women's hockey lead to players shooting from these areas more, as they may be contested differently relative to the NHL. While some published research in men's ice hockey has examined tactical structures using spatiotemporal data, the lack of data availability in women's hockey remains a limiting factor for analysis and interpretation of the current findings (12). Secondly, these findings could be explained by a form of survivorship bias, where women's hockey athletes and coaches may be influenced by the scoring trends exhibited in men's professional hockey. For example, athletes and coaches may see a high number of goals from the High Slot and High Circle areas in the NHL, and assume that these areas may provide equal value in women's ice hockey. Currently, limited published literature exists examining how tactical approaches are taught in women's hockey, though some evidence exists identifying that youth ice hockey coaches learn through mentorship and formal education programs (13, 14). However, it is uncertain if this mentorship and education is informed by appropriate and applicable data sources. Future research should examine specifically where coaches and women's ice hockey athletes learn from in order to better understand what influences player and coaching tactics. Given that the scoring and shooting rates differ as strongly as they do between women's and men's hockey, further examination is necessary to evaluate if changing offensive tactical approaches would lead to greater overall goal scoring in women's ice hockey.

Despite the differences seen in shooting behaviors, some important similarities existed between leagues. First, for each league, the greatest number of goals, and highest shooting percentages, came from the low slot and the slot. This finding is unsurprising, as research across multiple sports (ice hockey, soccer) have found that proximity to the net is the strongest predictor of goal scoring probability (2, 6). Secondly, the blueline wide zone featured the greatest number of shots across each league despite having one of the lowest shooting percentages for each respective league. This high shot volume is partly explained by this zone representing the largest geographical area, but likely also has tactical implications for each league. Collectively, when examining shooting and scoring behaviors, both similarities and distinct differences exist across leagues.

## Limitations

While this paper serves as an important first step in comparing and contrasting elite men's and women's ice hockey with sub-elite women's ice hockey, there are some limitations worth addressing. First, the comparison zones used in this study are adapted from professional men's hockey, and as such, may not be equally representative if they were created from women's professional data. Despite this and differences seen in shot conversion, both women's leagues showed some similarity in goal share in each zone, when taking into account the number of shots, that support the original design of these shooting zones created by WAR on ice. Further, the data used in this study examines shooting and scoring distributions at a general level, and did not include other variables that could influence scoring (e.g., shot velocity), or contextual information, such as if a shot was preceded by a pass crossing the midline, which has been shown to increase the probability of a shot resulting in a goal (15). Collectively, future work should examine contextual factors (e.g., score effects, team level effects), as well as other task constraints (e.g., defensive/offensive structures, and playing styles) to gather a more conclusive understanding of the differences and similarities across leagues. Lastly, it is important note that future research should explore these relationships with larger datasets across multiple seasons.

## Conclusion

Collectively, this work demonstrates likely differences in shooting and scoring trends across women's collegiate, women's professional, and men's professional hockey. Acknowledging how each league is uniquely successful is important, as normative data should be used to inform coaches’ pedagogical and tactical decisions. Clear opportunity exists for coaches and athletes who are willing to use league specific data to inform their offensive approach, while acknowledging that implementing tactical approaches from other leagues may not be applicable.

## Data Availability

The raw data supporting the conclusions of this article will be made available by the authors, without undue reservation.

## Footnotes

^1^ https://docs.google.com/spreadsheets/d/16GcIISv3FISAA7xuZNDV8glzQklZyDCDbPJYnOjymus

^2^ https://moneypuck.com/data.htm

^3^ http://blog.war-on-ice.com/new-defining-scoring-chances/index.html
